# Rationale and design of a prospective, observational study for the QUantitative EStimation of Thrombus burden in patients with ST-Elevation Myocardial Infarction using micro-computed tomography: the QUEST-STEMI trial

**DOI:** 10.1186/s12872-020-01393-5

**Published:** 2020-03-11

**Authors:** Efstratios Karagiannidis, Nikolaos V. Konstantinidis, Georgios Sofidis, Evangelia Chatzinikolaou, Georgios Sianos

**Affiliations:** 1 First Department of Cardiology, AHEPA University Hospital, Aristotle University of Thessaloniki, St. Kiriakidi 1, 54636 Thessaloniki, Greece; 2 Hellenic Centre for Marine Research, Former US Base at Gournes, 71003 Heraklion, Greece

**Keywords:** Thrombus, Thrombus aspiration, Micro-computed tomography, ST-elevation myocardial infarction, Interventional cardiology

## Abstract

**Background:**

Although the presence of thrombus in patients with ST-elevation myocardial infarction (STEMI) has been linked to adverse outcomes, routine thrombus aspiration has not been proven effective. A potential explanation is that these patients should be risk-stratified. Traditional clinical, laboratory and angiographic parameters used in clinical trials have been proven inadequate to classify patients. Aspirated thrombotic material characteristics might be an additional important parameter that has not yet been addressed. In this report, we aim to describe a methodological analysis of thrombus aspirated from coronary arteries during primary PCI using micro-Computed Τomography (micro-CT). These data will be combined with traditional factors to develop a risk-stratification system with high discriminative power for these patients.

**Methods:**

Eighty-seven patients with STEMI undergoing thrombus aspiration in AHEPA University Hospital, Greece, will be enrolled in the study. The first patient was enrolled in June 2018. After being aspirated, thrombi are preserved in formalin and their volume and density are calculated with micro-CT. Micro-CT allows us to create 3D models of thrombi from a series of x-ray projection images. These models are further analyzed to find the volume and density of extracted thrombi and to assess potential differences in their structure. Association of these variables with clinical parameters and angiographic outcomes will be explored.

**Discussion:**

QUEST-STEMI is-to our knowledge-the first study of volumetric coronary thrombus assessment by micro-CT. This method could be used in larger, clinically-oriented trials to help stratify patients with thrombus burden according to their risk for adverse outcomes.

**Trial registration:**

QUEST-STEMI trial ClinicalTrials.gov number: NCT03429608 Date of registration: February 12, 2018. The study was prospectively registered (registered prior to enrollment of the first participant).

## Background

### Introduction

Acute myocardial infarction with ST-elevation (STEMI) remains one of the leading causes of mortality, despite the advances in pharmacological therapy and in mechanical reperfusion therapy. It is attributed to acute thrombotic occlusion of an epicardial coronary artery due to rupture of an atherosclerotic plaque leading to platelet aggregation and thrombus formation. According to guidelines, the preferred reperfusion strategy is primary PCI, as it further reduces mortality compared to fibrinolytic therapy [1]. However, despite improving outcomes over time, mortality among patients with STEMI undergoing primary PCI remains significant [2]. Interestingly, the presence of thrombus in patients suffering from acute coronary syndrome and undergoing PCI has been found to increase the incidence of Major Adverse Cardiac and Cerebrovascular events (MACCE) [3]. Apart from MACCE, high thrombus burden in patients with STEMI has been independently associated with stent thrombosis [4], distal embolization [5], no-reflow phenomenon [6], late incomplete stent apposition and late stent thrombosis [7].

Conceptually, based on the evidence, mechanical removal of thrombus, using thrombus aspiration catheters, should be beneficial for the patients. However, large randomized controlled trials of routine thrombus aspiration (such as TAPAS [8], TASTE [9], TOTAL [10] and INFUSE-AMI [11]) showed controversial results, providing in total no evidence of distinct benefits for thrombus aspiration, according to many recent meta-analyses [12–15], Moreover, the TOTAL trial showed an increase in risk of stroke for patients undergoing thrombus aspiration [16]. Thus, according to the current guidelines of the European Society of Cardiology (ESC), routine thrombus aspiration is not recommended in patients with STEMI [17].

### Rationale for the study

A potential explanation for the controversial results of the randomized controlled trials could be that, in all these trials, thrombus aspiration was performed as a routine strategy in all patients presenting with STEMI, whereas these patients should be considered as a heterogeneous group and thus they should be risk-stratified. The need for patient-level risk stratification is also reflected in the recent guidelines of the ESC, according to which, although thrombus aspiration is not recommended as a routine strategy, it may be considered in certain patients [17]. However, no specification about the profile of patients, in whom thrombus aspiration should be undertaken, is provided, and performing thrombus aspiration is left at the discretion of the interventional cardiologist conducting the primary PCI. Presently, there is evidence suggesting that angiographically quantified thrombotic burden (as assessed using the classification by Gibson [18] and  by Sianos [19]) could potentially be one of the parameters affecting the clinical outcomes, as patients with large thrombus burden are at higher risk for future adverse outcomes [20]. Nonetheless, a subgroup analysis of the TOTAL trial, which assessed the benefit of thrombus aspiration in patients with high thrombus burden, showed that routine thrombus aspiration in this patient subgroup did not improve cardiovascular outcomes [21]. Hence, additional factors are required to stratify risk in patients with STEMI, since traditional clinical, laboratory and angiographic parameters used in clinical trials have been proven inadequate. Aspirated thrombotic material characteristics might be an additional important parameter that has not yet been addressed and might further provide evidence of patients that will benefit from thrombus removal.

Recent advances in non-invasive imaging enable the quantification of parameters that so far have only been subjective to the individual viewer. Micro-CT is an emerging non-destructive technique of 3D imaging, which has a much finer resolution compared to conventional Computational Tomography [22]. Although it was initially used for skeletal imaging, the development of contrast agents, which increase the low inherent contrast of soft-tissues in X-ray absorption, has expanded applications for micro-CT to the imaging of soft-tissues [22]. This facilitates precise soft-tissue visualization and characterization and can provide useful information about the compositional and morphological properties of extracted thrombi.

The purpose of the study is to assess for the first time, through the application of innovative technologies, important features of extracted thrombi, including their volume, their shape, their micro-architecture and their density, which might be linked to certain clinical outcomes. This will contribute to the development of a more sophisticated risk-stratification model with high discriminative power combining: clinical and laboratory data, angiographic parameters and data regarding the characteristics of aspirated thrombi, which will be derived using micro-CT. This might facilitate a more personalized risk-based approach in treating patients with STEMI.

## Methods/design of the study

### Study design and population

QUEST-STEMI (ClinicalTrials.gov Identifier: NCT03429608) is an investigator-initiated, prospective, single-arm, non-interventional cohort trial involving patients with STEMI, who undergo primary PCI and thrombus aspiration within 12 h of symptoms onset.

The trial is conducted in accordance with the principles set by the declaration of Helsinki, the International Conference on Harmonization Guidelines for Good Clinical Practice and all applicable regulatory requirements. The study protocol has been approved by the Medical Ethics Committee of the Aristotle University of Thessaloniki and by the Scientific Committee of AHEPA University Hospital. Each subject provides written informed consent before participating in the study.

A total of 87 patients presenting to AHEPA University Hospital with STEMI, who undergo primary PCI and thrombus aspiration at the discretion of the treating physician, will be enrolled in the study. Detailed eligibility criteria are described in Table 1.

**Table 1:** Summary of inclusion and exclusion criteria for the QUEST STEMI study

Inclusion criteria:	Exclusion criteria:
● Patients with symptoms of myocardial ischemia for at least 30 min ● ECG changes indicating STEMI ● Patients undergoing primary PCI and thrombus aspiration (at the discretion of the treating physician) within 12 h from symptom onset ● Written informed consent	● Patients who have received thrombolytic therapy for index STEMI event ● Known intolerance to heparin, aspirin or P2Y12 inhibitor therapy (clopidogrel, prasugrel, or ticagrelor)

The first patient was enrolled in QUEST-STEMI on 5 June 2018. As of December 2019, 70 patients have been enrolled. Completion of enrollment is anticipated in the first quarter of 2020. The results of the study are expected until the end of 2021.

### Thrombus aspiration procedure

The pharmacological treatment of each patient prior to PCI is according to standard practices (unfractionated heparin (100 IU/kg) and a loading dose of aspirin (325 mg) and either ticagrelor (180 mg) or prasugrel (60 mg) or clopidogrel (600 mg)) [17]. Thrombus aspiration is performed by experienced interventional cardiologists according to standard practices, as previously described [8–10]. Briefly, after crossing the lesion with a wire, the thrombus aspiration catheter is advanced proximal to the lesion. Two different aspiration systems, which are available at our institution, are being used: the 6F STENTYS aspiration catheter and the 6F Thrombuster II catheter (Kaneka). The choice of the aspiration device is at the discretion of the Interventional Cardiologist. Manual suction begins before the catheter crosses the lesion. The thrombus aspiration catheter is passed through the thrombotic occlusion many times, so that at least 40 cc of blood and material are aspirated. In case blood backflow stops suddenly during the procedure, the device should be removed to check for the presence of thrombus obstructing the lumen. The solid material aspirate is captured in a filter basket provided by the manufacturer of the thrombus aspiration catheter.

The aspirated thrombi are preserved in 10% formalin solution and are analyzed using the micro-CT.

### Micro-CT procedure

#### Staining

The samples are sent to the Biodiversity Laboratory of the Institute of Marine Biology, Biotechnology and Aquaculture (IMBBC) and are thoroughly washed with distilled water. Subsequently, they are subjected to dehydration procedures so that they are gradually stored in alcohol solution 70% (Metscher protocol) [23].

**Fig. 1:**
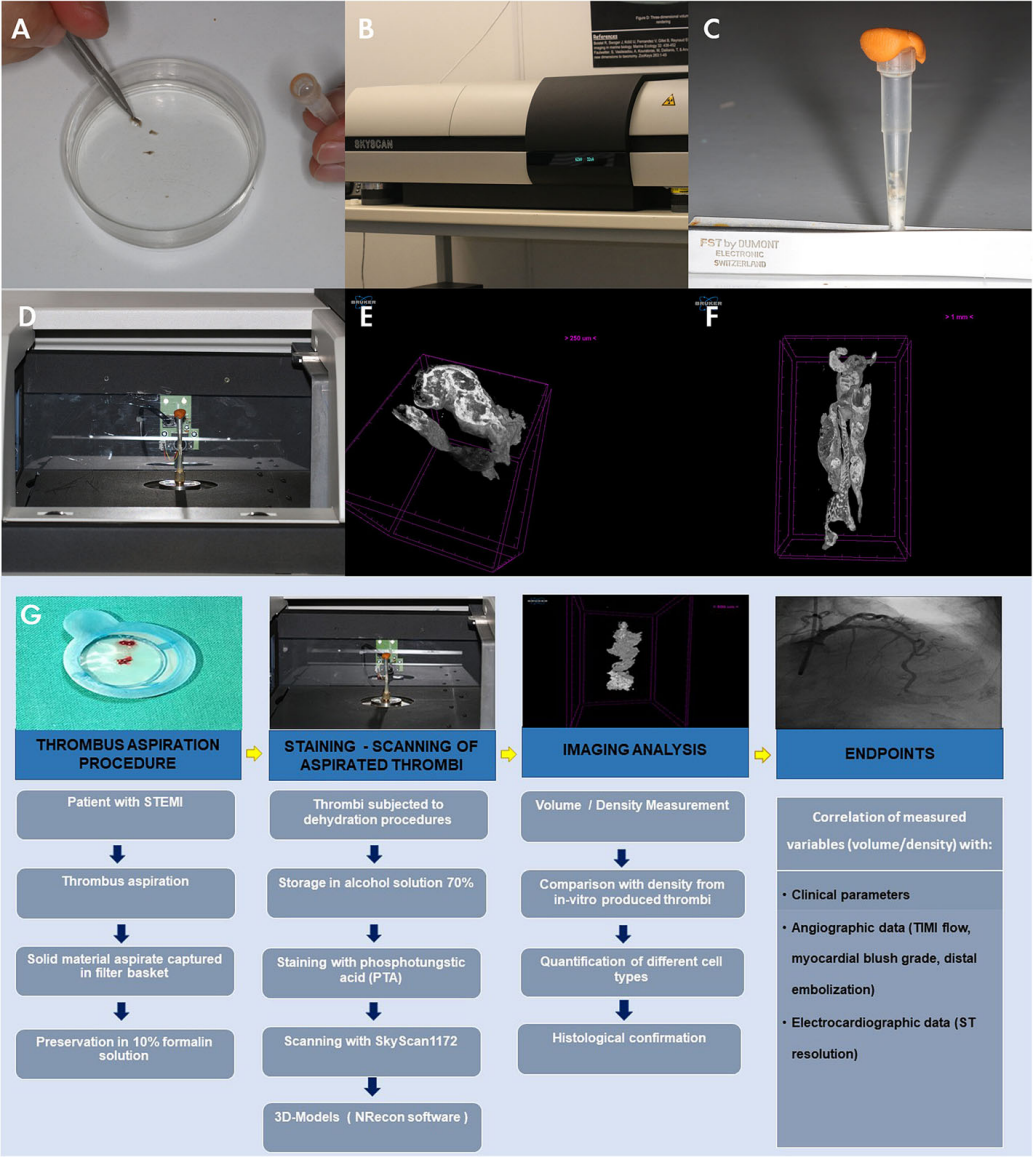
Methodology of the QUEST-STEMI study. **a** Staining of thrombi with phosphotungstic acid (PTA); **b** Skyscan 1172 in the Institute of Marine Biology, Biotechnology and Aquaculture Heraklion, Greece; **c** Samples are placed in a specific vial which contains ethanol; **d** Samples are mounted on the specific head inside the micro-CT; **e**-**f** Representative samples of thrombi. The samples were stained using 0.3% PTA as a contrast agent and scanned using Skyscan 1172 at a voltage of 48 kV and 204μΑ without filtering for a full rotation of 360^o^. Images were acquired at a pixel size of 5.52 μm with a camera binning of 1 × 1. The projections were reconstructed with the use of the NRecon (Bruker, Kontich, Belgium) software; **g** Graphical abstract-overview of the methodology of the QUEST-STEMI study

In order to achieve the best imaging of the thrombi, chemical factors which strengthen the contrast of tissues, such as phosphotungstic acid (PTA), are being used (Fig. 1a) to achieve best quality of the micro-CT tomographs [24]. This contrast factor is widely used in the Tomography because it binds to many proteins and to connective tissue.

#### Scanning

Thrombi are scanned by means of the micro-CT SkyScan 1172 in the Biodiversity lab of IMBBC (Fig. 1B). This system uses a tungsten source with energies ranging from 20 to 200 kV. It is equipped with an 11megapixel CCD camera (4000 × 2672 pixel) with a maximum analysis of < 0.8 μm/pixel.

After the staining of the clots with 0.3% PTA, every sample is placed in a specific vial which contains ethanol (Fig. 1C) and is mounted on the specific head inside the micro-CT (Fig. 1D). Subsequently, the principal parameters (e.g. voltage, magnification, resolution) are regulated to achieve the best imaging result. Scans are performed at a full rotation of 360°.

The scanning procedure results into a series of projection images arranged in the form of image stacks which, in turn, are reconstructed into sections (cross-section images) with the use of the NRecon (Bruker, Kontich, Belgium) software, which employs a modified Feldkamp’s back-projection algorithm. The resulting sections are combined to create the 3D models, which will be further analyzed to extract useful measurements for the shape characteristics of the thrombi, such as the volume and the relative density. All scans are reconstructed using the same range of attenuation coefficients (0–0.7) in order to obtain comparable results. Figure 1E-F shows two representative micro-CT images of extracted thrombi.

#### Imaging analysis

Shape analysis on the surface and internal structure of the three-dimensional representations of the clots will lead to the identification of important characteristics that will be used to estimate variability within samples and perform clustering for the substantial differences between thrombi. Those features would be ranging from measurements on the actual virtual models to analysis of parameter-values after the application of a parametric representation of the variability in tissue shape.

The calculation of the total clot volume is one of the intrinsic measurements arising from the 3D models and is performed using Amira software (VSG, Burlington USA).

Moreover, potential differences in clot internal and external structure (e.g. architecture of the various cell types) will be evaluated through the 3D models rendered from the image stacks.

The clot relative density is calculated with the CTAnalyser (CTAn, Bruker, Kontich, Belgium) software, using information from the variability and structure in the 3D gray scale values of the images.

Ιn-vitro produced thrombi, which are characterized by a known homogeneous composition, have also been scanned in order to identify the exact grey scale histogram values, which correspond to each type of tissue. These artificial clots have been constructed in vitro by mixing platelet enriched plasma and whole blood from healthy volunteers, as previously described [25]. In this way, samples consisting mainly of platelets (reminiscent of the “white thrombi”) and samples, which predominately consist of erythrocytes and which are expected to share the same properties with the naturally occurring “red thrombi”, have been formed. The mean values and standard deviations of densities have been determined from measured densities of eight samples for each type of thrombus and the cut-off Housefield Units of different tissue types in thrombi have been identified using threshold analysis. This enables tissue characterization by comparing the measured densities of extracted thrombi to the estimated densities of the homogeneous artificial thrombi. Thus, we are able to quantify the presence of different cell types (for instance erythrocytes and platelets) within a thrombus.

Analysis of the clots is performed by two independent researchers who are blinded to the clinical variables and the outcome of the patients.

### Histopathologic examination of thrombi

At the end of the experiment, histological and immunohistochemical analysis of thrombus aspirates is performed to validate the findings of the micro-CT analysis. Thrombi are embedded in paraffin blocks and cut into 4–5 μm sections. Routine staining is performed with hematoxylin and eosin. Moreover, thrombi are stained for leukocyte subsets, markers of thrombosis, and neutrophil extracellular traps (NETs), as described [26]. The stained sections are evaluated by two experienced pathologists, who are blinded to patient information. In each case, the percentage of fibrin, erythrocyte, and platelet area in relation to the whole thrombus area is investigated and thrombi are classified into white, mixed, and red categories according to their fibrin and erythrocyte content, as previously reported [25]. Furthermore, thrombi are classified according to their age into three groups: recent thrombi (< 1 day), lytic thrombi (1–5 days), and organized thrombi (> 5 days) [27].

### Endpoints- endpoint adjudication-patient follow-up

The study primarily aims to develop a methodology for the quantitative and qualitative assessment of aspirated thrombi, using micro-CT. Hence, important characteristics of thrombi, including their volume (in mm^3^) and their density (in Housefield units), will be measured. At a second level, correlation of these variables with clinical parameters, angiographic data and electrocardiographic data will be conducted. In particular, any association between the extracted thrombus volume and density with factors from patients’ medical history (e.g smoking, diabetes mellitus) will be explored. Moreover, potential correlation of the volume of aspirated thrombus burden, as assessed using the micro-CT and the angiographic classification of thrombus burden according to Sianos [19], will be examined. Furthermore, association of thrombus volume and thrombus density with electrocardiographic and angiographic outcomes suggestive of poor patient prognosis will be explored; in particular: ST-segment resolution (complete (> 70%), partial (30–70%), or absent (< 30%)) [28], post-procedural Thrombolysis in Myocardial Infarction(TIMI) flow and distal embolization. All patients will be followed-up (through telephone contacts on a quarterly basis) for 12 months for any MACCE (acute myocardial infarction, stent thrombosis, target lesion revascularization, cardiac death, cerebrovascular death, or stroke). Electrocardiographic and angiographic outcomes and MACCE will be evaluated based on pre-specified definitions by two experienced interventional cardiologists, who will be blinded to the findings of the micro-CT analysis. Detailed primary and secondary endpoints are described in Table 2.

**Table 2:** Primary and secondary endpoints for the QUEST STEMI study

**Primary endpoints**	
• Volume of aspirated thrombus burden (in mm^3^)	
• Density of aspirated thrombus burden (in Housefield Units)	
**Secondary endpoints**	
• Association between extracted thrombus volume/thrombus density and factors from patients’ medical history (diabetes mellitus, use of antiplatelet drugs or anticoagulants, pain-to-balloon time and history of smoking)	
• Correlation of the volume/density of aspirated thrombus burden with the Sianos’ classification of thrombus burden [19]	
• Association between extracted thrombus volume/density and ST-segment resolution. ST resolution will be classified as complete (> 70%), partial (30–70%), or absent (< 30%) [28]	
• Association between extracted thrombus volume/density and post-procedural Thrombolysis in Myocardial Infarction (TIMI) flow	
• Association between extracted thrombus volume/density and distal embolization	
• Association between extracted thrombus volume/density and myocardial blush grade	
• Association between extracted thrombus volume and angiographically evident residual thrombus burden	
• Association between extracted thrombus volume/density and the device used for thrombus aspiration	
• Association between extracted thrombus volume/density with MACCE (acute myocardial infarction, stent thrombosis, target lesion revascularization, cardiac death, cerebrovascular death, or stroke) at 12 months follow-up	

### Statistical considerations

#### Sample size estimation

Sample size estimation was performed using the G*Power tool [29]. The primary endpoint of the study is to develop a methodology for the quantitative and qualitative assessment of aspirated thrombi, using micro-CT. Thus, sample size estimation was based on a secondary endpoint of the study and in particular, on parameters affecting the volume of extracted thrombi. For the F-test, of the multivariate regression model using ten predictors for high thrombus burden and assuming power of 0.9, significance level of 0.05 and effect size of 0.3 (based on a preliminary analysis on the first 25 patients), the required number of subjects is 79. This initial sample size will be increased by 10%, because of the possibility that some patients might be lost to follow-up. Hence, we aim for a total sample size of 87 patients.

#### Statistical analysis

Continuous data will be presented as mean ± SD., whereas categorical variables will be displayed as counts. Normally distributed data will be analyzed using parametrical tests, such as Student t-test and for non-normally distributed data nonparametric Mann-Whitney test will be utilized. Categorical variables will be compared using chi-square test.

Intra- and interobserver variability of the measurements of the volume and the density of aspirated thrombi will be calculated to assess the reliability and the reproducibility of the proposed methodology. For this reason, a Bland–Altman analysis will be performed and the intraclass correlation coefficient (ICC) will be determined [30]. Regarding, qualitative variables Cohen’s k-coefficient will be calculated [31].

Univariate and multivariate linear and logistic regression analyses will be used to determine independent predictors of high thrombus burden and of adverse clinical outcomes. Receiver operating characteristic (ROC) curve analysis -and the derived area under the curve (AUC)- will be constructed to determine the optimal cut-off value for high and low thrombus burden. Cox regression analyses will be performed to identify independent predictors of MACCE. Kaplan-Meier survival will be calculated to evaluate the MACCE-free survival at 1 year follow-up, with the difference between different patient sub-groups analyzed by the log-rank test. Patients that are lost to follow-up will be censored at their last study encounter. Missing data in the multivariate model will be replaced by means of multiple imputation, using chained equations. To verify the stability of the results, complementary sensitivity analysis will be performed under various missing-data models. Statistical analysis will be performed using programming language R.

An overview of the design and the methodology of the QUEST-STEMI study is presented in Fig. 1 G.

## Discussion

In this study we aim to develop a methodology for the quantitative and qualitative assessment of extracted thrombi in patients presenting with STEMI and undergoing thrombus aspiration during primary PCI. Our goal is to quantify characteristics that so far have only been subjective to the individual viewer, such as the thrombus volume and the thrombus fine structure, and to investigate the predictive value of these characteristics.

These novel imaging parameters, combined with clinical and laboratory data, will enable the in-depth exploration of the complex cellular interactions in the STEMI setting and will potentially improve patient risk stratification. This might facilitate the development of a personalized approach in treating patients with STEMI.

Furthermore, the 2018 ESC / EACTS Guidelines on myocardial revascularization strongly support future trials of improved thrombus aspiration technologies in patients with high risk [17]. However, previous studies evaluating newly developed strategies for thrombus aspiration have failed to quantify the volume of thrombi retrieved [32]. Due to its high accuracy for the quantification of the volume of the extracted thrombotic material, this methodology will potentially enable the assessment of different thrombus aspiration technologies based on their ability for thrombus removal.

Micro-CT has often been used in medical studies [33, 34] and has been proven to accurately visualize and quantify venous thrombi in experimental models [35]. Nonetheless, this is-to our knowledge-the first study of volumetric coronary thrombus assessment by micro-CT. In the TOTAL-OCT study, a sub-study of the TOTAL trial, Optical Coherence Tomography (OCT) was used to quantify thrombus in vivo prior to stent implantation after restoration of TIMI 2–3 flow to the culprit vessel [36]. The study showed that measurement of pre-stent thrombus burden by OCT in patients suffering from STEMI is feasible and repeatable [36]. However, although intravascular OCT has a tremendous potential of helping us to visualize and manage thrombus in STEMI patients, this method has certain limitations: First, it cannot provide clear images in the thrombus-occluded vessel due to significant signal attenuation caused by erythrocytes [37]. Moreover, its shallow penetration depth (1.0 to 1.5 mm in a blood-free environment) might limit its ability to visualize the full size of a large clot [37]. Last, OCT cannot distinguish between mural thrombi and plaques [38]. On the other hand, micro-CT imaging provides the opportunity to use chemical factors which strengthen the contrast of tissues. This will facilitate an improved image quality of thrombi and will enable more accurate tissue characterization, as potential differences in clot internal and external structure, such as architecture of the various cell types, can be better visualized. Thus, OCT and micro-CT represent two complementary research pathways, which can both provide valuable information for the quantitative and qualitative characteristics of thrombi and for risk-stratification of patients with STEMI.

There are certain limitations to this study. First, the study is restricted to patients with STEMI who undergo thrombus aspiration at the discretion of the interventional cardiologist performing the primary PCI. Thus, the study population is not necessarily representative of the whole spectrum of STEMI patients. Moreover, additional imaging (OCT) will not be used to quantify residual thrombus, as this technology is not routinely available in our catheterization laboratory. Furthermore, it is possible that formalin fixation might influence the actual size of aspirated thrombi. This formalin-induced shrinkage effect might result in underestimating the actual dimensions of thrombi. Nonetheless, this will affect the size of all samples, as they are all subjected to the same fixation procedures. Last, the present study is a pilot study with small sample size, and it is not powered to show correlation between imaging variables and hard clinical endpoints.

In conclusion, we propose a novel methodological approach for the assessment of aspirated thrombotic material from coronary arteries using micro-CT, offering new perspectives for future research. This method could be used in larger, clinically-oriented trials to help stratify patients with thrombus burden according to their risk for adverse outcomes.

## Data Availability

Data are available from Georgios Sianos (e-mail: gsianos@auth.gr) upon reasonable request and with permission of AHEPA University Hospital.

## Abbreviations

ECG: Electrocardiogram
ESC: European society of cardiology
IMBBC: Institute of Marine Biology, biotechnology and aquaculture
MACCE: Major adverse cardiac and cerebrovascular events
Micro-CT: Micro-computed tomography
NETs: Neutrophil extracellular traps
OCT: Optical coherence tomography
PCI: Percutaneous coronary intervention
PTA: Phosphotungstic acid
STEMI: Myocardial infarction with ST-elevation
